# How Can We Develop an Efficient eHealth Service for Provision of Care for Elderly People with Balance Disorders and Risk of Falling? A Mixed Methods Study

**DOI:** 10.3390/ijerph18147410

**Published:** 2021-07-11

**Authors:** Andréa Gomes Martins Gaspar, Pedro Escada, Luís Velez Lapão

**Affiliations:** 1 Instituto de Higiene e Medicina Tropical (IHMT), Universidade NOVA de Lisboa (UNL), 1349-008 Lisbon, Portugal; luis.lapao@ihmt.unl.pt; 2 Hospital Beatriz Ângelo, 2674-514 Lisbon, Portugal; 3 Hospital Egas Moniz, 1349-019 Lisbon, Portugal; pedroalbertoescada@gmail.com

**Keywords:** balance disorders, falls, elderly care, eHealth, mixed methods

## Abstract

This study aimed to identify relevant topics for the development of an efficient eHealth service for elderly people with balance disorders and risk of falling, based on input from physicians providing healthcare to this patient group. In the quantitative part of the study, an open multiple-choice questionnaire was made available on the website of the Portuguese General Medical Council to assess the satisfaction with electronic medical records regarding clinical data available, the time needed to retrieve data and the usefulness of the data. Of the 118 participants, 55% were dissatisfied/very dissatisfied with data availability and 61% with the time spent to access and update data related to the focused patient group. Despite this negative experience, 76% considered future e-Health solutions as pertinent/very pertinent. Subsequently, these findings were further explored with eight semi-structured interviews. The physicians confirmed the reported dissatisfactions and pointed out the lack of comprehensive data and system interoperability as serious problems, causing inefficient health services with an overlap of emergency visits and uncoordinated diagnostics and treatment. In addition, they discussed the importance of camera and audio monitoring to add significant value. Our results indicate considerable potential for e-Health solutions, but substantial improvements are crucial to achieving such future solutions.

## 1. Introduction

### 1.1. The Burden of an Ageing Population: Portugal and the World

As is observed in other health systems [1,2,3,4,5,6,7], Portugal’s increasing life expectancy in recent decades has not been followed by an increase in healthy life years [4,6]. The prevalence of chronic diseases, comorbidities, disabilities and falls have increased with aging [1,2,3,4,5,6,7]. In fact, there are many causes for elderly people’s falls to happen, including age, environmental factors, inappropriate clothing and shoes, risky behavior, medications, and balance disorders [1,7,8,9].

Elderly falls represent an important public health problem, being the main cause of accidental death in the population over 65 years of age [1,8,9,10]. Although Portugal has one of the lowest rates of fall-related mortality in the elderly population of the Western European region, this issue has received attention from the Portuguese government [11].

**Figure 1:**
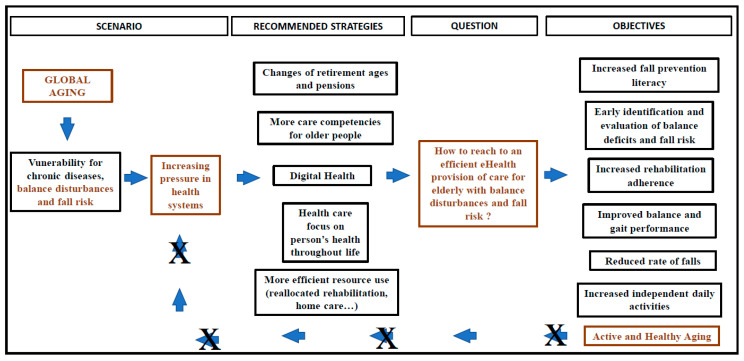
eHealth Framework for the elderly with balance disorders care provision. (Authors own elaboration).

The burden of aging with balance disorders and falls, and the insufficient access to healthcare data by health professionals, have led to additional medical visits, overdiagnosis, repeated diagnostic tests and multiple prescriptions [12,13,14,15]. This misuse of healthcare provision is costlier and unsustainable for the current healthcare provision model and considered unsuitable for responding to elderly population demand [4,12,13,14,15]. In order to relieve this pressure, new strategies have been recommended, including person-centered health systems and the utilization of devices and systems supported by Information Systems and Technologies (IST) [2,5,16] (Figure 1).

These healthcare solutions have revealed the potential to provide quality health services with complete, interoperable data in near real-time [17,18,19,20], and eHealth services with the engagement of patients and families for self and remote management of chronic conditions and prevention of risky behaviors [2,20,21,22]. Indeed, many researchers have studied the potential of digital sensors to identify early balance deficit and identify fallers among elderly people, improving the data quality of clinical tests and functional scales as the Timed Up and Go Test (TUGT) and the Berg Balance Scale [23,24,25,26,27,28,29]. Other researchers have evaluated the benefits of eHealth devices in balance training, reducing the risk of falling [29,30,31]. The eHealth services seem to have the potential to be a complementary method for preventive monitoring of falls, telerehabilitation, and monitoring of effective rehabilitation for elderly with balance disorder and risk of falling [23,24,25,26,27,28,29,30,31], aligned with the eHealth definition: “*an emerging field in the intersection of medical informatics, public health and business, referring to health services and information delivered or enhanced through the Internet and related technologies. In a broader sense, the term characterizes not only a technical development, but also a state-of-mind, a way of thinking, an attitude, and a commitment for networked, global thinking, to improve health care locally, regionally, and worldwide by using information and communication technology*” [32]. However, there are still constraints to be overcome: technological obsolescence; unsuitable technological devices; regulation, standardization, auditing, inspection and quality control; lack of interoperability; health professional resistance; low organizational capability for new ways of working and organization; shortage of digital training [33,34,35,36,37,38]. In Europe, both the skill development in old age and the aging of younger generations of technology users have led to a growing number of elderly people able to use health and care services online, although to a lesser extent in Portugal [39].

### 1.2. Portugal and the Strategies Supported by IST

To build up a shared ecosystem of health information, the Portuguese Ministry of Health created an Electronic Health Record (EHR) called “Sclínico,” which is unfortunately not yet available in all health units of the Portuguese National Health Service (NHS) [40]. More recently, another digital service was made available on the NHS’ digital platform to allow the sharing of clinical information between all levels of health care and to promote the interaction between the citizen and the family health unit [40]. In addition, the Portuguese elderly people can use the current telephone and digital service of the NHS Call Center, known as “SNS 24,” which is responsible for the triage of first-level emergencies and guiding the population about health problems [40]. Another NHS telephone service, known as Senior Proximity Project, was implemented to identify the risks and needs of elderly people to reduce morbidity and promote more autonomy and health literacy [41]. Several public health units have provided retinal examination by teleradiology, teleconsultation, telediagnostic-telepathology, telemonitoring of cardiac and pulmonary diseases and telerehabilitation of osteoarticular disease of shoulder and knee [40]. Additionally, the electronic prescription system and the treatment guide for the user have allowed patients, including elderly people with chronic diseases, to obtain their medication without ever going to a health care unit [40].

However, in recent reviews [23,24,25,26,27,28,29,30,31], it was pointed out that the clinical applicability of eHealth devices and services in screening, assessing and treating elderly people with balance disorders and the risk of falling in Portugal is still unknown. Therefore, we aim at studying how to obtain an efficient eHealth service for the provision of care for elderly people with balance disorders and the risk of falling.

The purposes of this explanatory sequential mixed methods approach [42] were: (a) to identify and understand how to overcome the medical difficulties about availability of clinical data in the electronic medical record (EMR) relatively to the context of healthcare provision for elderly with balance disorders and risk of falling; (b) to know and understand the medical relevance about eHealth services to support health care for elderly people with balance disorders and risk of falls; (c) to understand how to develop an efficient eHealth service to support health care for elderly people with balance disorders and risk of falls.

The increasing interest of elderly people in medical digital devices and the eHealth potential to enhance health promotion and physician–patient interaction to mitigate care access inequities and to allow remote management of balance disorders and risk of falling are opportunities that should be further explored for active and healthy aging [29]. This could be viewed as an opportunity to mitigate the aging pressure in the health systems.

## 2. Materials and Methods

### 2.1. Study Design

**Figure 2:**
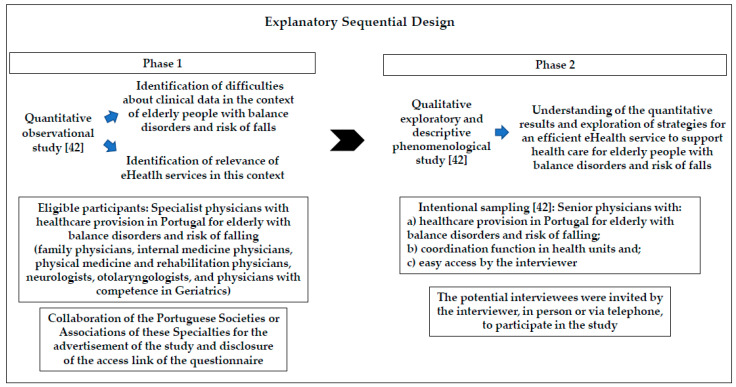
Design of the mixed methods study performed.

From June to August 2019, the authors performed a quantitative observational descriptive study [42] to identify the difficulties about clinical data and the relevance of eHealth (Figure 2).

This first phase aimed at responding to the following specific research questions: “Do physicians have difficulties accessing current clinical data in EMR relatively to the context of healthcare provision for elderly with balance disorders and risk of falling?”; “In this context, what is the medical satisfaction level with the use (e.g., time spent to access and fill in clinical data) and quality (e.g., availability of sufficient and understandable clinical data) of the current clinical data in the electronic medical record (EMR)?”, and “Could eHealth services be relevant to improve healthcare?”.

From December 2019 to April 2020, a qualitative exploratory and descriptive phenomenological study [42] was performed to understand the quantitative results and how to obtain an efficient eHealth service to support health care for elderly people with balance disorders and risk of falls. This second phase explored the following research questions: “What are the medical difficulties related with current clinical data in the context of health care provision for elderly with balance disorders and risk of falling?”; “What strategies can be implemented to improve clinical data?”; “What do you think about the contribution of eHealth?”; “How can an eHealth service be suitable? What are the necessary strategies? What difficulties must be overcome?”.

### 2.2. Materials

The questionnaire, entitled “Health contribution to the provision of health care for the elderly at risk of falling due to balance disorders” (in the original: “A contribuição do eHealth na prestação de cuidados de saúde ao idoso com risco de queda por distúrbios do equilíbrio”) was developed with 18 multiple choice questions [42]. It included socio-demographic data of the participants, availability of data in the EMR and relevance of eHealth in the context of health care for the elderly with balance disorder and risk of falling. Except for the demographic questions, alternative responses on quantity, frequency and evaluation were used, and a non-response (“Do not know/Do not answer”) was provided [42] (see Table A1). The usability, technical functionality and time to complete the questionnaire were tested. The access link was available through the website of the Portuguese General Medical Council (Ordem dos Médicos de Portugal), the entity that regulates medical practice in Portugal (https://ordemdosmedicos.pt/inquerito-a-contribuicao-do-ehealth-na-prestacao-de-cuidados-de-saude-ao-idoso/ (accessed on 25 June 2019)). The information regarding this open survey was provided online. The eligible participants were specialist physicians who provide healthcare in Portugal for the elderly with balance disorders and risk of falling, including family physicians, internal medicine physicians, physical medicine and rehabilitation (PMR) physicians, neurologists, otolaryngologists and physicians with competence in Geriatrics. For the advertisement of the study and disclosure of the access link of the questionnaire, the authors requested, via email, the collaboration of the Portuguese Society of Physical Medicine and Rehabilitation, Portuguese Association of General and Family Medicine, Portuguese Society of Internal Medicine, Center for Geriatric Studies of the Portuguese Society of Internal Medicine, Portuguese Society of Otorhinolaryngology and Portuguese Otoneurology Association. The Portuguese Society of Neurology was also contacted through this institution’s website. The questionnaire was distributed online using the survey software SurveyMonkey^®^ [43]. Each question was made available in turn, with the possibility of returning to the previous questions. All questions had one mandatory answer [44]. During the study time, the IP address of the participants was used to eliminate potential duplicate responses from the same user [44].

Regarding the qualitative study, the same interviewer (one of the authors) conducted individual semi-structured interviews [42]. Four primary thematic categories were discussed: current clinical data in the context of health provision for elderly with balance disorders and risk of falling (i.e., understanding of quantitative results), interventions to improve the clinical data, understanding of eHealth relevance pointed out by physicians in the quantitative research, and strategies to improve the use of eHealth services (see Table A2). The sampling was intentional [42], with a purposeful search for physicians with: (a) healthcare provision for elderly with balance disorders and risk of falling; (b) coordination function in health units and; (c) easy access by the interviewer. The potential interviewees were invited by the interviewer, in person or via telephone, to participate in the study. The participant number was defined after saturation or redundancy of responses; that is, the sampling process was completed when no new information emerged from the new interviews [42]. Respecting the anonymous participation of the quantitative study, the interviewer did not ask if the interviewee had participated in the previous study.

### 2.3. Data Analysis

Firstly, to determine the quantitative frequency tables [42], a descriptive and exploratory statistics of the data from the questionnaires were performed. The software IBM SPSS Statistics version 26 (IBM Corporation, Armonk, NY, USA) was used [45]. Secondly, a descriptive analysis of demographic data [42] of interviewed was performed. All the interviews were manually coded and transcribed by the interviewer, allowing content analysis of interviews [42]. For a better comprehension of the quotes, the authors entered words in round brackets.

### 2.4. Ethical Considerations

The survey’s aim was clearly identified in both the website of the Portuguese General Medical Council and on the SurveyMonkey^®^ link [SURVEY PREVIEW MODE] A contribuição do eHealth na prestação de cuidados de saúde ao idoso com risco de queda por distúrbios do equilíbrio Survey (surveymonkey.com, accessed on 25 June 2019). The physicians could voluntarily participate and leave the study until the submission of the questionnaire. The information of the quantitative study was treated confidentially and anonymously by using respondent e-mails confidentiality and anonymous responses features of the software SurveyMonkey^®^ [43].

Regarding the interviews, the participants signed a consent form and received a copy of this and information about the study. They could leave the study until one month after the interview’s date. The audio recording was authorized by the participants. To guarantee confidentiality, all interviews were manually coded. The transcriptions omitted information to avoid identifying respondents. All data were kept anonymous [42]. The information from the questionnaires and interviews and the audio records were kept in a safe place (external disk with access code) within the period provided by the Portuguese law [46], always safeguarding the confidentiality of the information obtained.

## 3. Results

### 3.1. First Phase: Quantitative Research

The online questionnaire had a total of 118 responses. This represents 1% of the total universe of 12,214 [47] family physicians, internal medicine physicians, PMR physicians, neurologists and otolaryngologists registered in Portugal (Table 1).

There was no duplicate response found with the same IP address. A relevant proportion of the respondent activity was directed to provide care to elderly people in the context studied. About the elderly people observed by physicians, 19% of the participants said that their monthly appointment time was more than 50% occupied with elderly patients with balance disorders, while 9% of physicians had their monthly appointment time more than 50% occupied with elderly patients with complaints related to consequent falls. A total of 86% of the physicians recognized the relevance of data about the previous provision of health care to the elderly with balance disorders and risk of falling. However, A total of 43% of all physicians responded that they need to access data from previous care consultations for elderly patients with balance disorder and risk of falling in more than half of cases. The majority of the participants (84%) had access to this information through the hospital or health center electronic medical record. Most respondents (60%) reported that more than half of the medical consultation time had been spent on IST-related activities. Moreover, 50% of participants were dissatisfied or very dissatisfied with the use of IST (e.g., usefulness, quality) in the context of balance disorders and the risk of falling in the elderly.

#### 3.1.1. Socio-Demographic Participant Data

Most of the participants were female, accounting for 72 responses (61%). Younger physicians adhered more to the study: most participants (74%) had 50 years old or less. Although 64% of the eligible physicians were over 50 years old (7787 out of 12,214), the participants over 50 years old represented only 26% (31 out of 118) of the responses.

About 39% of the participants were specialists in Internal Medicine, 38% in Otolaryngology, 15% were family physicians, 4% PMR physicians, and 3% were neurologists. Only 4% were enrolled in the College of Competence in Geriatrics. Comparing the numbers, the family physicians had weak participation (18 out of 7451 family physicians), although being the specialty most represented among the eligible physicians.

Most physicians (82%) had the main job in public healthcare units, and 54% were from the larger Portuguese health region, the Lisbon and Tejo Valey (LTV) Regional Health Administration. (Table 1)

#### 3.1.2. Difficulties and Medical Satisfaction Level Related to Current Clinical Data in the EMR (Electronic Medical Registration)

61% of all respondents were dissatisfied or very dissatisfied with the time spent accessing clinical data in the EMR, rising to 65% when considering only professionals who have the main job in a public healthcare institution. Regarding the availability of sufficient and understandable clinical data in the EMR, 55% of the physicians revealed dissatisfaction or a lot of dissatisfaction, with values of 59% for public health professionals as their main job. Again, 61% of all participants also expressed dissatisfaction or great dissatisfaction with time spent to fill in new data in the EMR, reaching 64% among professionals with the main job in a public health institution (Table 2).

#### 3.1.3. Relevance of the Use of eHealth

The possibility of using eHealth for elderly patients with balance disorder and risk of falling was considered pertinent or very pertinent by 76% of all physicians and also by professionals with public healthcare as the main job. Regarding the medical specialties with more than 30 responses, 72% (33 out of 46) and 82% (37 out of 45) of internal medicine physicians and otolaryngologists, respectively, considered remote services as pertinent or very pertinent. If we consider only the participants of Internal Medicine and Otolaryngology working in the public sector as their main job, the percentages remain at 72% (31 out of 43) and rise to 86% (25 out of 29), respectively (Table 3).

### 3.2. Second phase: Qualitative Research

The same interviewer conducted a total of seven face-to-face semi-structured interviews and one semi-structured interview by mobile phone due to coronavirus pandemic limitations. This interview phase was limited to senior physicians who provided healthcare to the elderly, with different training in technology and medical experience.

#### 3.2.1. Socio-Demographic Participant Data

Five male and three female physicians, aged 47–66 years old, participated in the study. Two were family physicians, two internal medicine physicians, one neurologist and three otolaryngologists. All of them were either graduated or senior consultants. Six physicians were coordinators in their public health units, and two were coordinators of private otoneurology units. One physician was from the Regional Health Administration of the center (Center) of Portugal, and the others were from Lisbon and Tejo Valey (LTV) (Table 4).

#### 3.2.2. Content Analysis

As mentioned previously, four primary thematic categories were discussed. Twelve subthemes emerged from data analysis (Table 5).

The interviewees pointed out some misuse of healthcare provision by the elderly people in Portugal, meaning using above what is necessary of emergency visits, drug therapies and complementary diagnostic tests: *The elderly Portuguese population has no specific education on how to access healthcare services properly* (Participant 5). They also agreed on the need to access complete data: *… the elderly people often represent complex patients… the intervention … requires multiple specialties …* (Participant 1).

Relative to the medical dissatisfaction with available clinical data identified in the quantitative research, all interviewees highlighted the lack of a comprehensive data set and the lack of interoperability of computer systems: *We* (Physicians) *get to know more or less the drugs that are prescribed … We don’t know more …* (Participant 4); *I have asked them* (family physicians) *to send me information. So, I can get a sense of what is going on with the patient.* (Participant 8); *... the data records are, sometimes, incomplete, they are not very explicit* (Participant 1); *The computer systems … have great incompatibilities with each other. … because the operating systems are different, or because the internet browser is different.* (Participant 5).

Some interventions to improve clinical data were pointed out: more investments in the interoperability of health information systems and in the organization of work with time for remote interaction and consultation: *Medicine will have to be a Medicine of shared information*. (Participant 1); *We should have some time allocated for this* (remote consultations). *So that we can keep our head on it and we will be really effective*. (Participant 4). Regarding the use of eHealth services for elderly people with balance disorders and the risk of falling, only Interviewee 2 questioned its proper applicability, justifying the doubts due to his lack of experience: *… I don’t know how this* (remote health care provision) *is done at a distance … I don’t even see myself doing a thing like that … I think the physician-patient relationship is something that is impossible to be computerized.* (Participant 2). The other physicians, similar to most of the participants of the quantitative research, considered the eHealth contribution to be beneficial: *… it* (eHealth) *could be a great help because vertigo has many decompensations…* (and) *they* (patients) *are afraid of being … without connection to the physician.* (Participant 7).

The interviewees pointed out the potential benefits of eHealth as a complementary channel to healthcare: rational use of resources with lower pressure on hospital resources, more healthcare access, better communication between medical specialties, closer physician–patient relation and more participation of patient and caregiver at home. However, they only agreed with the eHealth use for a follow-up consultation. The age was not considered a limitation for eHealth use. About the improvement of the use of eHealth services, the interviewees mentioned the need for more discussions to address the essential parameters for remote interaction and the need for involving eHealth system managers and programmers: *… it is crucial the collaboration between the technology and those responsible for the technology…* (Participant 6). In addition, the need for availability of clinical and interactive data and the motivation for human involvement were mentioned.

As essential strategies, the participants considered the inclusion of medications in use, the analyzes and imaging tests, and the registration of activities of daily living [48]. The use of questionnaires, calendars and graphics of trends on the occurrence of balance disorders and falls, supported by physician and patient’s records, was also considered as a closer way of managing the disease: *… simple questions like “Have you had a fall last year?”, “Was there any injury? Yes, are you afraid of falling due to this injury?* (Participant 5); *… interactive questionnaires…, for example, in the recurrent vertigo… to have documented how many episodes …what kind of triggers…* (Participant 7). Warning messages for adverse effects of medications or falls were classified as beneficial. The availability of individual balance exercises with a checklist and the possibility of uploading patient videos for clinical follow-up were other issues discussed: *…the patient could record what they are feeling, for example, eye movements and then they uploaded the recording … We* (physicians) *could include some exercises on the platform …*

The interviewees mentioned the relevance of camera and audio for monitoring of balance rehabilitation and closer interaction, especially in cases of gait assessment and depression: *… a phone call is one thing. If there is a camera it may even allow you* (physician) *to see, for example, the patient’s gait…* (Participant 3); … *to monitor through videos, through cameras, as long as the patient gives his consent, of course …* (Participant 7).

The eHealth service management by a physician was considered essential: *When there are changes* (in the health)*, the physician can also be consulted.* (Participant 3); *…* *always a physician*. (Participant 7).

In addition, the security of using eHealth was discussed: …what type of password one (physician) should use … encrypted… addressed to the clinical team with security code (Participant 5).

The participants also referred to the relevance of active motivation and involvement of patient and caregiver in disease prevention and control: *… Patients cannot continue to think that the responsibility of their health belongs to the physician… the patients have to be involved and responsible for their health…* (Participant 7); *…we often think that our elderly people do not have the ability to manage new technologies … but we can have a caregiver who can contact us remotely…* (Participant 5) Finally, the investment in medical awareness and eHealth training was highlighted, allowing better physician involvement: *… this is a work to be done in medical education … after some time of implementation* (of medical education)*, I am convinced that it* (eHealth) *will be the future of medicine…* (Participant 1).

## 4. Discussion

A mixed-methods study was performed to know how to develop an efficient eHealth service for the provision of care for elderly people with balance disorders and the risk of falling, i.e., the research problem. Our findings revealed negative experiences with EMR, contributing to a misuse of the health care system. Despite this, the highlighted relevance of eHealth in this matter is an incentive for the development of future solutions.

Unfortunately, we had a low response number in the quantitative research, as described in other studies [49,50]. This limited the comparison between the specialties and between public or private health provision groups. Despite this, we could confirm the presence of constraints regarding the data availability in Portugal. We observed medical dissatisfaction with the information systems in general. In total, 50% of the participants of our study were dissatisfied or very dissatisfied with the current usefulness and quality of IST in the context of balance disorders and the risk of falling in the elderly. The physicians were dissatisfied or very dissatisfied with available data in EMR and time spent to access and update clinical data. According to the interviews, the incomplete or not understandable information about medical consultations and the lack of integration of clinical data between the health units have contributed to the misuse of healthcare provisions by elderly people, with multiple consultations, repeated prescriptions, polypharmacy and increasing costs that could be minimized with an appropriate digital service. These limitations and consequent costs have been reported by other authors [4,12,13,14,15]. The participants pointed out the need for investments in the interoperability of health information systems and in the organization of work to overcome this situation, as previously proposed in other studies [19,20,21]. In fact, data are essential for healthcare provision, monitoring of population health status and decision making. To reach real-time universal data in healthcare, interoperability issues should be addressed.

As in previous studies [2,5,16], our findings also confirmed the relevance of the use of eHealth services. However, the current way of working and interacting with patients should be restructured, including dedicated time to interact digitally with patients. eHealth can be leveraged as a complementary method to provide healthcare services, including preventive monitoring of falls and telerehabilitation with evaluation and monitoring of balance diseases and falls. For the interviewed participants, the remote consultation or management should be only for follow-up consultations, and it cannot fully replace the face-to-face clinical evaluations. As mentioned by Catan et al. [34], face-to-face consultations reduce anxiety whenever people need a physician. Only one interviewed revealed skepticism about digital solutions due to the lack of eHealth clinical experience. Several studies have already revealed the influence of limited knowledge about telemedicine on the perception of the potential of eHealth [19,35].

Finally, we confirmed the need for improvement of eHealth services for a more effective healthcare provision. The qualitative research allowed exploring interventions to achieve an efficient eHealth service to support healthcare for elderly people with balance disorders and risk of falling. Several suggestions were pointed out: the inclusion of complete clinical data, the possibility for interactive communication, message alerts and remote availability of balance exercises. Camera and audio were considered essential elements for closer interaction, allowing remote viewing of the gait, as well as the balance exercises performed. All the parameters should be aligned between technology experts and physicians to design suitable technological services. eHealth services and devices should be user-friendly and suitable for both the health professional and for the patient.

For the participants, the physician emerged as the main manager of eHealth service, but not necessarily the only one [51]. As in other studies [35,37], physicians also highlighted the need for investment for confidentiality and security of data. This should always be ensured. Relatively to human resources, strategies to motivate, educate and train the elderly patient and caregiver were also discussed. Self-care and self-management of health and disease (e.g., promotion of health and prevention of disease) should be further encouraged [2,5,16]. The need for health professional awareness and training to use all of the potential of digital solutions were mentioned, including the investment in professional health education. Thus, the potential of digital health could be widely used with motivated and trained human resources [2,20]. The Portuguese health system should be adjusted to tackle aging demand, overcoming the constraints of the EHR and the lack of interoperability of the information systems. The implementation of a universal digital health coverage system supported by comprehensive digital tools with camera and audio resources can better contribute to active and healthy aging [52] with more efficient management of health care for elderly with balance disorder and risk of falling. The design and the development of a balance disorder-related remote service, with the recommended functionalities, is an opportunity worth to be explored. The strategies identified and discussed in this study will be fed into a Design Science [50] process to design and implement a future eHealth service for a more effective provision of healthcare for elderly with balance disorders at a distance.

### Limitations

Regarding the participants of the web-based questionnaire, we should acknowledge possible selection biases [53] of the quantitative research. Despite the intention to recruit physicians of different specialties, a small participant size was observed as in other online studies. This made it difficult to compare the results between specialties and to know if there is a difference between the public or private health provision groups. Another consideration, already highlighted in other web-based surveys, is the age of the respondents. Most participants of this research were younger physicians who seem to have more technological resources and online interests and to be more receptive to web-based questionnaires. In addition, the higher percentage of responses from otolaryngologists can be explained by their focus of interest in inner ear diseases that can promote balance disorders. The family physicians had weak participation (only 18 out of 7451), although this specialty represented most of the eligible participants. Due to the reduced or null number of responses from geographically more remote areas (e.g., Azores), we could not analyze and, in the second phase of the mixed methods study, explore more deeply the data of these participants that could most benefit from the potential of eHealth.

Additionally, the multiple-choice questions of the questionnaire allowed an easier analysis, but this approach did not allow the inclusion and discussion of supplementary opinions of the respondents.

Relatively to the qualitative research, the sampling was intentional. The last interview was conducted via mobile phone due to the limitation of the coronavirus pandemic.

The population targeted in both studies was limited to physicians with the provision of outpatient health care to the elderly in Portugal.

All these facts limited the generalizability of the findings.

## 5. Conclusions

Despite significant obstacles in existing digital solutions, 76% of the Portuguese physicians included in this study considered future e-Health services as highly relevant for complementary healthcare for elderly people with balance disorders and the risk of falling. The use of eHealth services comprised of digital technologies such as cameras, sensors and audio monitoring may reinforce such solutions. Additionally, these services may represent considerable potential for reducing the excess of emergency visits, and the overlap of drug therapies and diagnostic procedures and improved treatment. This may increase both health care efficiency and quality and contribute to relieving pressure in the escalating health care costs. However, significant constraints regarding the current availability of clinical data in EHR care systems were described. The insufficient quality of both available data in EMR and in the time needed to access such data and to register new clinical data was stated. We would like to highlight that our study group’s description of incomplete, or not understandable, information about medical consultations and the lack of integration of clinical data indicate serious challenges to overcome. More research about this topic is also required to further enhance the knowledge about the use of digital tools in this field of health care.

## Figures and Tables

**Table 1:** Study quantitative: Demographic data of the participants. PMR: physical medicine and rehabilitation.

Demographic Data/Specialty	Family Physician	Internal Medicine Physician	PMR Physician	Neurologist	Otolaryngologist	Total
Physician number according to Portuguese General Medical Council—year 2019 [47]	7451	2847	691	549	676	12,214
Number of participants of the study according to specialty (%)	18 (15.3%)	46 (39.0%)	5 (4.2%)	4 (3.4%)	45 (38.1%)	118 (100%)
Participation according to specialty total number of physicians (%)	18/7451 (0.2%)	46/2847 (1.6%)	5/691 (0.7%)	4/549 (0.7%)	45/676 (6.7%)	118/12 214 (1.0%)
Participant’s gender (M/F)	3/15	15/31	3/2	1/3	24/21	46/72
Participant age ≤ 50 years old/Total physician number age ≤ 50 years old ^a^	15/2390	34/1299	5/271	3/234	30/233	87/4427
Participant age >51 years old/Total physician number age > 51 years old ^a^	3/5061	12/1548	0/420	1/315	15/443	31/7787
Regional Health Administration of Portugal						
-North	2	13	1	0	8	24
-Center	0	7	1	0	12	20
-Lisbon and Tejo Valey	16	19	3	4	22	64
-Alentejo	0	3	0	0	1	4
-Algarve	0	4	0	0	2	6
-Madeira	0	0	0	0	0	0
-Azores	0	0	0	0	0	0
Main job—Public sector	17	43	4	4	29	97
Main job—Private sector	1	3	1	0	16	21

^a^ According to Portuguese General Medical Council (“Ordem dos Médicos de Portugal”)—year 2019 [47].

**Table 2:** Questionnaire: Satisfaction degree with clinical data in the EMR—Context of health care provision for elderly with balance disorders and risk of falling.

Satisfaction Degree/Specialty	Family Physician	Internal Medicine Physician	PMR Physician	Neurologist	Otolaryngologist	Total
Time to data access (public and private main job)						
-S	1	12	3	1	25	42 (36%)
-D	17	34	1	3	17	72 (61%)
-Others	0	0	1	0	3	4 (3%)
TOTAL	18	46	5	4	45	118 (100%)
Time to data access (public main job)						
-S	1	11	3	1	15	31 (32%)
-D	16	32	0	3	12	63 (65%)
-Others	0	0	1	0	2	3 (3%)
TOTAL	17	43	4	4	29	97 (100%)
Sufficient/understandable data (public and private main job)						
-S	3	18	3	1	25	50 (42%)
-D	15	27	2	3	18	65 (55%)
-Others	0	1	0	0	2	3 (3%)
TOTAL	18	46	5	4	45	118 (100%)
Sufficient/understandable data (public main job)						
-S	3	16	3	1	15	38 (39%)
-D	14	26	1	3	13	57 (59%)
-Others	0	1	0	0	1	2 (2%)
TOTAL	17	43	4	4	29	97 (100%)
Time to fill data (public and private main job)						
-S	2	13	2	0	22	39 (33%)
-D	16	29	3	4	20	72 (61%)
-Others	0	4	0	0	3	7 (6%)
TOTAL	18	46	5	4	45	118 (100%)
Time to fill data (public main job)						
-S	2	12	2	0	13	29 (30%)
-D	15	27	2	4	14	62 (64%)
-Others	0	4	0	0	2	6 (6%)
TOTAL	17	43	4	4	29	97 (100%)

S: Satisfied or very satisfied. D: Dissatisfied or very dissatisfied. Others: Did not use or Did not answer or Did not know.

**Table 3:** Questionnaire: Relevance degree about the use of eHealth in the context of care provision for the elderly with balance disorders and the risk of falling.

Relevance of eHealth / Specialty	Family Physician	Internal Medicine Physician	PMR Physician	Neurologist	Otolaryngologist	Total
Public and private main job						
-Pertinent	13	33	5	2	37	90 (76%)
-No pertinent	2	3	0	1	3	9 (8%)
-Indifferent	2	5	0	0	3	10 (8%)
-Others	1	5	0	1	2	9 (8%)
TOTAL	18	46	5	4	45	118 (100%)
Public main job						
-Pertinent	12	31	4	2	25	74 (77%)
-No pertinent	2	3	0	1	1	7 (7%)
-Indifferent	2	4	0	0	2	8 (8%)
-Others	1	5	0	1	1	8 (8%)
TOTAL	17	43	4	4	29	97 (100%)

Others: Did not answer or Did not know.

**Table 4:** Study qualitative: Socio-demographic data of the participants and interview features.

Participant	Gender	Age	Specialty	Regional Health Administration of Portugal	Main Job	Interview	Audio Recording
1	M	59	Otolaryngology	LTV	Public sector	Face-to-face	Y
2	M	55	Neurology	LTV	Public sector	Face-to-face	Y
3	M	53	Internal Medicine	LTV	Public sector	Face-to-face	Y
4	M	59	Internal Medicine	LTV	Public sector	Face-to-face	Y
5	M	47	Family Medicine	LTV	Public sector	Face-to-face	Y
6	F	66	Family Medicine	Center	Public sector	Face-to-face	Y
7	F	49	Otolaryngology	LTV	Private sector	Face-to-face	Y
8	F	55	Otolaryngology	LTV	Private sector	Mobile phone	Y

M: Male; F: Female; LTV: Lisbon and Tejo Valey.

**Table 5:** Thematic categories of the qualitative research.

Thematic Categories
1. Current clinical data in the context of health provision for elderly with balance disorders and risk of falling: understanding of the medical dissatisfaction identified in the quantitative research 1.1. Availability 1.2. Barriers
2. Interventions to improve the clinical data 2.1. Interoperability of computer health systems 2.2. New work organization
3. eHealth contribution in this context: understanding of the relevance observed in the quantitative research 3.1. eHealth benefits
4. Strategies to improve eHealth services for a more effective healthcare provision 4.1. Clinical and interactive data 4.2. Audiovisual technology 4.3. eHealth management 4.4. Security of eHealth use 4.5. Motivation and training of patient 4.6. Caregiver involvement 4.7. Medical training

## Data Availability

The data that support the findings of this study are available on request from the corresponding author, AGMG.

## Appendix A

**Table A1:** Quantitative study: answer choices of the questionnaire.

Number Question	Subject	Answer Choices
Q1	Sex	Male Female
Q2	Age	= or <30 31–40 41–50 51–60 = or >61
Q3	Specialty	Physical Medicine and Rehabilitation General and Family Medicine Internal Medicine Otorhinolaryngology Neurology
Q4	Competence in Geriatrics	Yes No
Q5	Main job	Personalized public health care unit Family health unit Public hospital Hospital in public-private partnership University hospital Private health unit
Q6	Regional Health Administration	North Center Lisbon and Tejo Valey Alentejo Algarve Madeira Azores
Q7	Monthly frequency of health care provision for elderly with balance disorders	= or <25% 26 a 50% 51 a 75% =or >76% Do not answer/Do not know
Q8	Monthly frequency of health care provision for elderly with consequent falls	= or <25% 26 a 50% 51 a 75% = or >76% Do not answer/Do not know
Q9	Need to access data from previous care consultations of elderly people with balance disorders and risk of falling	= or <25% 26 a 50% 51 a 75% = or >76% Do not answer/Do not know
Q10	Access to data from previous care consultations for elderly people with balance disorders and risk of falling	Clinical paper process Electronic medical record (EMR) Paper information provided by the patient Do not answer/Do not know
Q11	Relevance of data about previous health care to the elderly with balance disorders and risk of falling for a new provision of healthcare in this context	Never Rarely Sometimes Often Always Do not answer/Do not know
Q12	Estimated time spent on Information Systems and Technologies (IST)-related activities	= or >76% 51 a 75% 26 a 50% = or <25% Do not use Do not answer/Do not know
Q13	General usefulness of clinical data in the EMR	Excellent Very good Good Bad Very bad Do not use Do not answer/Do not know
Q14	Satisfaction with time spent to access clinical data, in the EMR, from previous care consultations for elderly people with balance disorders and risk of falling	Very dissatisfied Dissatisfied Satisfied Very Satisfied Do not use Do not answer/Do not know
Q15	Satisfaction with availability of sufficient and understandable clinical data, in the EMR, from previous care consultations for elderly people with balance disorders and risk of falling	Very dissatisfied Dissatisfied Satisfied Very Satisfied Do not use Do not answer/Do not know
Q16	Satisfaction with time spent to fill-in new data, in the EMR, related to the provision of health care to the elderly with balance disorders and risk of falling	Very dissatisfied Dissatisfied Satisfied Very Satisfied Do not use Do not answer/Do not know
Q17	General satisfaction with the use of IST (usefulness, quality) in the context of elderly with balance disorders and risk of falling	Very dissatisfied Dissatisfied Satisfied Very Satisfied Do not use Do not answer/Do not know
Q18	Relevance of eHealth in the context of elderly with balance disorders and risk of falling	Very relevant Relevant No difference Irrelevant Very irrelevant Do not answer/Do not know

## Appendix B

**Table A2:** Qualitative study: Interview guide.

Thematic Categories	Questions
Current clinical data in Portugal	“What are the medical difficulties related with current clinical data in the context of health care provision for elderly with balance disorders and risk of falling?”
Interventions to improve the clinical data	What strategies can be implemented to improve clinical data?
eHealth contribution	“What do you think about the contribution of eHealth?”
Strategies to improve the use of eHealth services	How can eHealth services be suitable? What are the necessary strategies? What difficulties must be overcome?

## References

[1] European Innovation Partnership on Active and Healthy Ageing (EIP-AHA) (2013). Action Plan on Specific Action on Innovation in Support of Personalized Health Management, Starting with a Falls Prevention Initiative. https://ec.europa.eu/eip/ageing/library/action-plan-specific-action-innovation-support-personalized-health-management-starting-falls_en.html.

[2] The International Society for Quality in Health Care (ISQua) (2016). Health Systems and Their Sustainability: Dealing with the Impending Pressures of Ageing, Chronic and Complex Conditions, Technology and Resource Constraints. Whitepaper. https://isqua.org/resources-blog/resources?page=1&search=Health%20Systems%20and%20their%20Sustainability&date_range_start=&date_range_end=.

[3] National Institute for Health and Care Excellence (NICE) (2016). Multimorbility: Clinical Assessment and Management. https://www.nice.org.uk/guidance/ng56.

[4] Simões J., Augusto G.F., Fronteira I., Hernández-Quevedo C. (2017). Portugal: Health system review. Health Syst. Transit..

[5] Amalberti R., Vincent C., Nicklin W., Braithwaite J. (2019). Coping with more people with more illness. Part 1: The nature of the challenge and the implications for safety and quality. Int. J. Qual. Health Care.

[6] OECD (2019). Health at a Glance 2019: OECD Indicators.

[7] European Union (2020). Ageing Europe—Looking at the Lives of Older People in the EU. https://ec.europa.eu/eurostat.

[8] Salzman B. (2010). Gait and balance disorders in older adults. Am. FAM Physician.

[9] Health Evidence Network (HEN)—WHO (2004). What are the Main Risk Factors for Falls Amongst Older People and What are the Most Effective Interventions to Prevent These Falls?. http://www.euro.who.int/document/E82552.pdf.

[10] Ha V.A.T., Nguyen T.N., Nguyen T.X., Nguyen H.T.T., Nguyen T.T.H., Nguyen A.T., Pham T., Thanh Vu H.T. (2021). Prevalence and Factors Associated with Falls among Older Outpatients. Int. J. Environ. Res. Public Health.

[11] Haagsma J.A., Olij B.F., Majdan M., van Beeck E.F., Vos T., Castle C.D., Dingels Z.V., Fox J.T., Hamilton E.B., Liu Z. (2020). Falls in older aged adults in 22 European countries: Incidence, mortality and burden of disease from 1990 to 2017. INJ Prev..

[12] Kerber K.A. (2009). Vertigo and Dizziness in the Emergency Department. Emerg. Med. Clin. N. Am..

[13] Heinrich S., Rapp K., Rissmann U., Becker C., König H.H. (2010). Cost of falls in old age: A systematic review. Osteoporos. Int..

[14] Tehrani A.S.S., Coughlan D., Hsieh Y.H., Mantokoudis G., Korley F.K., Kerber K.A., Frick K.D., Newman-Toker D.E. (2013). Rising Annual Costs of Dizziness Presentations to U.S. Emergency Departments. Acad. Emerg. Med..

[15] Reis L., Lameiras R., Cavilhas P., Escada P. (2016). Epidemiology of Vertigo on Hospital Emergency. Acta Med. Port..

[16] Nolte E., Merkur S., Anell A. (2020). Achieving Person-Centred Health Systems.

[17] Winter A., Takabayashi K., Jahn F., Kimura E., Engelbrecht R., Haux R., Honda M., Hübner U.H., Inoue S., Kohl C.D. (2017). Quality Requirements for Electronic Health Record Systems. A Japanese-German Information Management Perspective. Methods Inf. Med..

[18] Ammenwerth E., Duftschmid G., Al-Hamdan Z., Bawadi H., Cheung N.T., Cho K.H., Goldfarb G., Gülkesen K.H., Harel N., Kimura M. (2020). International Comparison of Six Basic eHealth Indicators Across 14 Countries: An eHealth Benchmarking Study. Methods Inf. Med..

[19] Lapão L.V., Dussault G. (2017). The contribution of eHealth and mHealth to improving the performance of the health workforce: A review. Public Health Panorama..

[20] Blandford A. (2019). HCI for health and wellbeing: Challenges and opportunities. Int J. Hum. Comput. Stud..

[21] World Health Organization (WHO) (2017). mHealth Use of Appropriate Digital Technologies for Public Health—EB142/20. https://apps.who.int/gb/ebwha/pdf_files/EB142/B142_20-en.pdf.

[22] Uei S.L., Kuo Y.M., Tsai C.H., Kuo Y.L. (2017). An Exploration of Intent to Use Telehealth at Home for Patients with Chronic Diseases. Int. J. Environ. Res. Public Health.

[23] Sun R., Sosnoff J.J. (2018). Novel sensing technology in fall risk assessment in older adults: A systematic review. BMC Geriatr..

[24] Rucco R., Sorriso A., Liparoti M., Ferraioli G., Sorrentino P., Ambrosanio M., Baselice F. (2018). Type and Location of Wearable Sensors for Monitoring Falls during Static and Dynamic Tasks in Healthy Elderly: A Review. Sensors.

[25] Nguyen H., Mirza F., Naeem M.A., Baig M.M. (2018). Falls management framework for supporting an independent lifestyle for older adults: A systematic review. Aging Clin. Exp. Res..

[26] Leirós-Rodríguez R., García-Soidán J.L., Romo-Pérez V. (2019). Analyzing the Use of Accelerometers as a Method of Early Diagnosis of Alterations in Balance in Elderly People: A Systematic Review. Sensors.

[27] Montesinos L., Castaldo R., Pecchia L. (2018). Wearable Inertial Sensors for Fall Risk Assessment and Prediction in Older Adults: A Systematic Review and Meta-Analysis. IEEE Trans. Neural Syst. Rehabil. Eng..

[28] Bet P., Castro P.C., Ponti M.A. (2019). Fall detection and fall risk assessment in older person using wearable sensors: A systematic review. Intern. J. Med. Inform..

[29] Gaspar A.G.M., Lapão L.V. (2021). eHealth for Addressing Balance Disorders in the Elderly: Systematic Review. J. Med. Internet Res..

[30] Skjæret N., Nawaz A., Morat T., Schoene D., Helbostad J.L., Vereijken B. (2016). Exercise and rehabilitation delivered through exergames in older adults: An integrative review of technologies, safety and efficacy. Int. J. Med. Inf..

[31] Choi S.D., Guo L., Kang D., Xiong S. (2017). Exergame technology and interactive interventions for elderly fall prevention: A systematic literature review. Appl. Erg..

[32] Eysenbach G. (2001). What is e-health?. J. Med. Internet Res..

[33] Poenaru C., Poenaru E., Vinereanu D. (2014). Current Perception of Telemedicine in an EU Country. Maedica.

[34] Catan G., Espanha R., Mendes R.V., Toren O., Chinitz D. (2015). Health information technology implementation—Impacts and policy considerations: A comparison between Israel and Portugal. ISR J. Health Policy Res..

[35] Ayatollahi H., Sarabi F.Z.P., Langarizadeh M. (2015). Clinicians’ Knowledge and Perception of Telemedicine Technology. Perspect Health Inf. Manag..

[36] Radhakrishnan K., Xie B., Berkley A., Kim M. (2016). Barriers and Facilitators for Sustainability of Tele-Homecare Programs: A Systematic Review. Health Serv. Res..

[37] Albarraka A.I., Mohammedb R., Almarshoudc N., Almujalli L., Aljaeed R., Altuwaijiri S., Albohairy T. (2021). Assessment of physician’s knowledge, perception and willingness of telemedicine in Riyadh region, Saudi Arabia. J. Infect. Public Health.

[38] Wynn R., Gabarron E., Johnsen J.K., Traver V. (2020). Special Issue on E-Health Services. Int. J. Environ. Res. Public Health.

[39] Gil H. (2019). The elderly and the digital inclusion: A brief reference to the initiatives of the European union and Portugal. MOJ Gerontol. Ger..

[40] SPMS, Portuguese National Centre of Telehealth (CNTS) National Strategic Telehealth Plan (PENTS) 2019–2022. https://www.spms.min-saude.pt/wp-content/uploads/2019/11/PENTS_Tradução.pdf.

[41] SPMS SNS 24 Projeto Proximidade Senior. https://www.spms.min-saude.pt/pesquisa-geral/?_sf_s=idoso.

[42] Creswell J.W., Creswell J.D. (2018). Research design. Qualitative, Quantitative, and Mixed Methods Approaches.

[43] SurveyMonkey. https://help.surveymonkey.com/articles/en_US/kb/How-do-I-make-surveys-anonymous.

[44] Eysenbach G. (2004). Improving the quality of Web surveys: The Checklist for Reporting Results of Internet E-Surveys (CHERRIES). J. Med. Internet Res..

[45] IBM SPSS Statistics. https://www.ibm.com/products/spss-statistics/details.

[46] Assembleia da República Lei nº 58/2019 de 8 de Agosto de 2019. Diário da República n.º 151/2019, Série I de 2019-08-08. 3-40. https://data.dre.pt/eli/lei/58/2019/08/08/p/dre.

[47] Ordem dos Médicos de Portugal Estatísticas 2019. https://ordemdosmedicos.pt/wp-content/uploads/2020/01/ESTATISTICAS_ESPECIALIDADES_2019.pdf.

[48] WHO Active Ageing, A Policy Framework. A Contribution of the WHO to the Second United Nations World Assembly on Ageing, Madrid, Spain, April, 2002. https://extranet.who.int/agefriendlyworld/wp-content/uploads/2014/06/WHO-Active-Ageing-Framework.pdf.

[49] Aitken C., Power R., Dwyer R. (2008). A very low response rate in an on-line survey of medical practitioners. Aust. N. Z. J. Public Health.

[50] Lapão L.V., Da Silva M.M., Gregório J. (2017). Implementing an online pharmaceutical service using design science research. BMC Med. Inform. Decis. Mak..

[51] Lapão L.V., Pisco L. (2019). Primary health care reform in Portugal, 2005–2018: The future and challenges of coming of age. Cad. Saude Publica.

[52] Liotta G., Canhão H., Cenko F., Cutini R., Vellone E., Illario M., Kardas P., Poscia A., Sousa R.D., Palombi L. (2018). Active Ageing in Europe: Adding Healthy Life to Years. Front. Med..

[53] Eysenbach G., Wyatt J. (2002). Using the Internet for surveys and health research. J. Med. Internet Res..

